# The Deep Ocean's Carbon Exhaust

**DOI:** 10.1029/2021GB007156

**Published:** 2022-07-21

**Authors:** Haidi Chen, F. Alexander Haumann, Lynne D. Talley, Kenneth S. Johnson, Jorge L. Sarmiento

**Affiliations:** ^1^ Atmospheric and Oceanic Sciences Program Princeton University Princeton NJ USA; ^2^ Scripps Institution of Oceanography University of California, San Diego La Jolla California USA; ^3^ Monterey Bay Aquarium Research Institute Moss Landing CA USA

**Keywords:** carbon cycle, ocean circulation, Southern Ocean, biogeochemistry

## Abstract

The deep ocean releases large amounts of old, pre‐industrial carbon dioxide (CO_2_) to the atmosphere through upwelling in the Southern Ocean, which counters the marine carbon uptake occurring elsewhere. This Southern Ocean CO_2_ release is relevant to the global climate because its changes could alter atmospheric CO_2_ levels on long time scales, and also affects the present‐day potential of the Southern Ocean to take up anthropogenic CO_2_. Here, year‐round profiling float measurements show that this CO_2_ release arises from a zonal band of upwelling waters between the Subantarctic Front and wintertime sea‐ice edge. This band of high CO_2_ subsurface water coincides with the outcropping of the 27.8 kg m^−3^ isoneutral density surface that characterizes Indo‐Pacific Deep Water (IPDW). It has a potential partial pressure of CO_2_ exceeding current atmospheric CO_2_ levels (∆PCO_2_) by 175 ± 32 μatm. Ship‐based measurements reveal that IPDW exhibits a distinct ∆PCO_2_ maximum in the ocean, which is set by remineralization of organic carbon and originates from the northern Pacific and Indian Ocean basins. Below this IPDW layer, the carbon content increases downwards, whereas ∆PCO_2_ decreases. Most of this vertical ∆PCO_2_ decline results from decreasing temperatures and increasing alkalinity due to an increased fraction of calcium carbonate dissolution. These two factors limit the CO_2_ outgassing from the high‐carbon content deep waters on more southerly surface outcrops. Our results imply that the response of Southern Ocean CO_2_ fluxes to possible future changes in upwelling are sensitive to the subsurface carbon chemistry set by the vertical remineralization and dissolution profiles.

## Key Points

- Deep ocean CO_2_ release arises from high potential CO_2_ partial pressure in subsurface water between the Subantarctic Front and sea‐ice edge
- Maximum subsurface potential CO_2_ is connected to the outcrop of upwelling Indo‐Pacific Deep Water (IPDW), rich in remineralized organic carbon
- High alkalinity and low temperature limit CO_2_ release from higher carbon‐content deep water outcropping south of IPDW

## 1 Introduction

**Figure 1:**
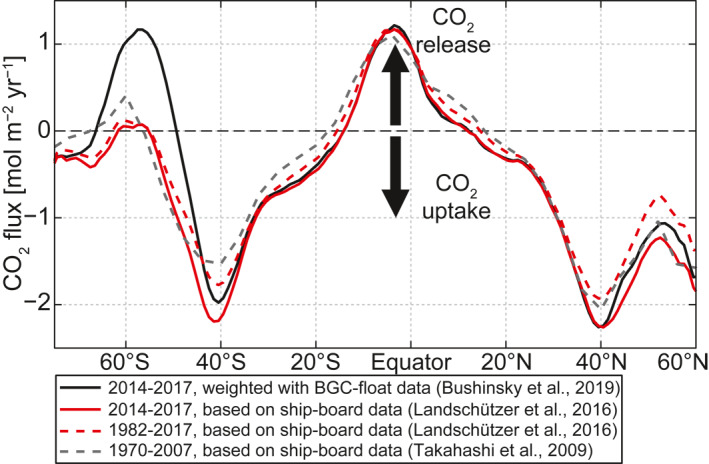
Zonal and annual mean global ocean to atmosphere CO_2_ flux (positive values denote ocean CO_2_ release to the atmosphere) from different data products for different time periods. Black: Neural‐network estimate, weighted with biogeochemical Argo float data (Bushinsky et al., 2019; Landschützer et al., 2019). Red: Neural‐network estimate, based on ship‐board observations from the SOCAT database (Landschützer et al., 2016, 2020). Gray: Climatology based on ship‐board observations from the CDIAC database (Takahashi et al., 2009).

Based on year‐round biogeochemical measurements with profiling floats that resolve the seasonal cycle, recent work has identified a larger than previously estimated release of carbon dioxide (CO_2_) from the Southern Ocean to the atmosphere during austral winter (black line in Figure 1; Bushinsky et al., 2019; Gray et al., 2018). While there has been a broad consensus that the region south of about 50°S releases old, pre‐industrial CO_2_ to the atmosphere (Gruber et al., 2019; Mikaloff Fletcher et al., 2007; Morrison et al., 2015; Wu et al., 2019), the magnitude and extent of this CO_2_ release have been substantially smaller in previous estimates, which were derived from ship‐board measurements (gray and red lines in Figure 1; Landschützer et al., 2016, 2020; Takahashi et al., 2009). These previous estimates most likely underestimate the CO_2_ release due to a lack of data during austral winter. Therefore, analysis of data that are unaffected by seasonal biases is required to better understand the Southern Ocean's potential to release CO_2_. In this study, we analyze ship and float measurements from depths that are not subject to large seasonal variations in order to address the question of whether such a large outgassing from the Southern Ocean during winter can be expected when deep waters are brought to the surface.

In the Southern Ocean (south of about 50°S), CO_2_ is naturally released from the ocean to the atmosphere due to the upwelling of carbon‐rich waters from the deep layers of the global ocean (Mikaloff Fletcher et al., 2007; Morrison et al., 2015; Takahashi et al., 2009) that elevate the surface dissolved inorganic carbon (DIC) content in this region (Wu et al., 2019). Globally, this Southern Ocean CO_2_ release is balanced by natural CO_2_ uptake in the subtropics and northern subpolar oceans (Figure 1; Gruber et al., 2009; Takahashi et al., 2002, 2009). This CO_2_ uptake is driven in part by cooling of the surface water as it is being transported to higher latitudes, which increases its capacity to hold CO_2_, and in part by the sinking of biologically produced organic matter out of the surface layer (Gruber et al., 2009; Takahashi et al., 2002, 2009). The biologically produced organic carbon sinks through the thermocline and is remineralized at depth, where it feeds the deep waters with inorganic carbon, which is then transported to the Southern Ocean (Sarmiento & Gruber, 2006). Therefore, the upwelling and CO_2_ release in the Southern Ocean closes the global ocean carbon cycle.

The leakage of old, pre‐industrial CO_2_ from the deepest ocean layers is unique to the high‐latitude Southern Ocean, because this is the only region where deep waters ascend to the surface along rising, steep isopycnals (Morrison et al., 2015; Speer et al., 2000; Talley, 2013; Toggweiler & Samuels, 1995). Deep waters exit the Indian, Pacific and Atlantic basins in both western and eastern boundary currents, and spiral southeastward and upward until reaching the base of the mixed layer in the southern Antarctic Circumpolar Current (ACC; Tamsitt et al., 2017, 2019). This upwelling process is thought to be the major return pathway for remineralized carbon from the ocean's interior to the surface (Kwon et al., 2009; Sarmiento et al., 1988; Skinner et al., 2010). Estimates of ocean transport show that a supply of DIC to the Southern Ocean surface comes from a southward and upward flux of Circumpolar Deep Water (Iudicone et al., 2011; Wu et al., 2019). This southward transport consists of North Atlantic Deep Water (NADW), Lower Circumpolar Deep Water (LCDW) in the Indian and Pacific basins, and somewhat less dense Indo‐Pacific Deep Water (IPDW) that all carry DIC from sinking and remineralization of biogenic carbon, and, in the case of NADW, DIC from air‐sea gas exchange (Aldama‐Campino et al., 2020; Broecker & Peng, 1992).

The upwelling of the DIC‐rich waters as well as the low surface temperature are responsible for a strong meridional surface DIC increase toward the high latitudes, as recently shown by Wu et al. (2019). Moreover, the study shows that an elevated Southern Ocean DIC is maintained by a larger capacity of the water to hold carbon due to the cold conditions and elevated alkalinity (Alk) associated with the upwelled waters. In contrast to DIC that is high throughout most of the Southern Ocean surface waters (Wu et al., 2019), the CO_2_ release is concentrated to a relatively narrow band between about 50° and 65°S (Figure 1). The latter is driven by an elevated surface ocean partial pressure of CO_2_ (*p*CO_2_) over atmospheric *p*CO_2_ ($p{CO}_{2}^{atm}$), whereas the ocean's *p*CO_2_ is set by the seawater properties such as DIC, Alk, temperature, and salinity. Therefore, a more detailed understanding of the *p*CO_2_ of the upwelling deep waters is required to explain the deep ocean's CO_2_ release through Southern Ocean upwelling.

In this study, we aim to further our understanding of the underlying mechanisms of this deep ocean CO_2_ release in the Southern Ocean and to identify its sources. We seek to explain the characteristic meridional patterns of surface CO_2_ fluxes in the Southern Ocean (Figure 1), to explain why CO_2_ release peaks in a zonal band between 55° and 65°S, and to identify the pathway and sources of how the deep ocean loses carbon to the atmosphere. For this purpose, we assess factors that could affect the deep ocean's outgassing potential using subsurface DIC, Alk, dissolved oxygen, nutrient, temperature, and salinity data from ship‐based observations (Key et al., 2004, 2015; Lauvset et al., 2016; Olsen et al., 2016) and biogeochemical floats (Johnson et al., 2017). In order to relate these ocean observational data to the Southern Ocean surface CO_2_ fluxes, in a first step, we here make use of the interior ocean potential *p*CO_2_ (PCO_2_; Broecker & Peng, 1982; Skinner et al., 2010) excess above the approximate current (year 2015) global‐mean $p{CO}_{2}^{atm}$ (400 μatm), that is, ∆PCO_2_ = PCO_2_ − $p{CO}_{2}^{atm}$. Thus, we calculate the seawater PCO_2_ as the *p*CO_2_ that a water parcel would attain if it were adiabatically brought to the surface (Broecker & Peng, 1982) using its DIC, alkalinity, potential temperature (θAl) and sea‐level pressure. The resulting ∆PCO_2_ is then a more accurate measure of the capability of upwelling water to release CO_2_ to the present‐day atmosphere than DIC alone. In a second step, we analyze the drivers of the spatial patterns of ∆PCO_2_ in the interior ocean by estimating the different contributions of the dissolution, solubility, ocean circulation and ventilation, and biological carbon pump processes (Gruber & Sarmiento, 2002; Sarmiento & Gruber, 2006; Volk & Hoffert, 1985). In a third step, we decompose ∆PCO_2_ vertical gradient into components associated with DIC, temperature, and Alk. Using this decomposition, we demonstrate the importance of considering the full vertical carbon chemistry structure, in particular Alk, when assessing the influence of the carbon‐rich deep ocean on the surface Southern Ocean CO_2_ fluxes.

## 2 Methods

### 2.1 Potential *p*CO_2_ and Neutral Density

Potential *p*CO_2_ (PCO_2_) is defined as the *p*CO_2_ that a water parcel would attain if it was brought to the surface adiabatically (Broecker & Peng, 1982; Sarmiento & Gruber, 2006; Skinner et al., 2010), thus correcting for the pressure effects on temperature and partial pressure. PCO_2_ is computed following Williams et al. (2017) using CO2SYS (van Heuven et al., 2011) and by using dissociation constants of carbonate from Lueker et al. (2000), of sulfate from Dickson (1990), and of fluoride from Perez and Fraga (1987), and the boron to salinity ratio of Lee et al. (2010). Here, PCO_2_ is a function of Alk, DIC, potential temperature (*θ*, referenced to 0 dbar), practical salinity (S), the reference pressure (p^ref^ = 0 dbar), silicate (Si), and phosphate (PO_4_). For biogeochemical float data (Section 3), we use nitrate (NO_3_) and convert it to PO_4_ and Si using stoichiometric phosphate‐to‐nitrate and silicate‐to‐nitrate ratios of 1:16 and 2.5, respectively (Anderson & Sarmiento, 1994). Note that we here use the deviation of the interior ocean PCO_2_ from the approximate current (year 2015) atmospheric *p*CO_2_ of 400 μatm (∆PCO_2_), which differs from past or future atmospheric conditions, to illustrate the present‐day outgassing potential of an interior ocean water masses.

We here evaluate the interior ocean ∆PCO_2_ structure considering neutral density surfaces. For this purpose, we have calculated the neutral density (*γ* ^n^) for all data sets (Section 3) based on Jackett and McDougall (1997; version 3.05.12; 17 June 2019; http://www.teos-10.org/preteos10_software/neutral_density.html). Since in this code *γ* ^n^ is not defined for temperatures below −2.5°C, the small set of temperatures below this value is set to −2.5°C.

### 2.2 Temperature and Salinity Effects on ∆PCO_2_

In a first step, we estimate the effect of dilution of ocean tracers through freshwater fluxes on PCO_2_ ($∆{PCO}_{2}^{dil}$) by evaluating the difference between PCO_2_ and sPCO_2_ derived from salinity normalized (s) quantities:

(1) $$\begin{array}{l} ∆PCO_{2}^{dil}=PCO_{2}-sPCO_{2}. \end{array}$$

Here, sPCO_2_ is derived using sAlk, sDIC, sSi, and sPO_4_, whereas salinity normalization of a given variable *X* to a reference salinity (S^ref^ = 34.7) is performed through (Broecker & Peng, 1992; Chen & Millero, 1979)

(2) $$\begin{array}{l} sX=\frac{X}{S}S^{ref}. \end{array}$$

The only exception here is sDIC, which we obtain by first salinity normalizing its preindustrial value and then adding the anthropogenic component (see Section 3).

In a second step, we estimate the effect of solubility on the interior ocean PCO_2_ distribution ($∆{PCO}_{2}^{sol}$) through

(3) $$\begin{array}{l} ∆PCO_{2}^{sol}=sPCO_{2}-PCO_{2}^{DIC,Alk}. \end{array}$$

Here, ${PCO}_{2}^{DIC,Alk}$ is the PCO_2_ arising from variations in sDIC and sAlk and it is computed as a function of sAlk, sDIC, *θ* ^ref^ (2.5°C), S^ref^ (34.7), p^ref^ (0 dbar), sSi, and sPO_4_. Reference values *θ* ^ref^ and S^ref^ are arbitrary values that depend on the purpose of the analysis (Table 1). The resulting $∆{PCO}_{2}^{sol}$ provides a measure for the contribution of mostly temperature differences relative to the location of the reference value. Since we are interested in the ocean interior structure of PCO_2_, we chose these reference values to reflect deep water properties. If for example, *θ* ^ref^ was set to 20°C, which is closer to global surface water temperatures, the interior ocean ${PCO}_{2}^{DIC,Alk}$ would become much larger than the actual PCO_2_ and $∆{PCO}_{2}^{sol}$ strongly negative everywhere. We note that these solubility effects on PCO_2_ should not be confused with solubility effects on DIC through air‐sea exchange in response to surface heat fluxes, that is, the so‐called solubility pump. We further note that the separation of the DIC and Alk into the pump components (Sections 2.3 and 2.4) is not affected by the choice of *θ* ^ref^.

**Table 1:** Reference Values Used in This Study for the Estimation of the Drivers of Interior Ocean PCO_2_ Variations, in Part Derived From Gridded GLODAPv2 Data (Lauvset et al., 2016)

Variable	Value	Description
p^ref^	0 dbar	Reference pressure
S^ref^	34.7	Reference ocean salinity
*θ* ^ref^	2.5°C	Reference ocean temperature
${pCO}_{2}^{atm}$	400 µatm	Approximate atmospheric partial pressure of CO_2_ in 2015
${PCO}_{2}^{ref}$	131 µatm	Partial pressure of CO_2_ using reference values of this table
sAlk^ref^	2,298 μmol kg^−1^	Reference surface ocean alkalinity
${sPO}_{4}^{ref}$	0.1 μmol kg^−1^	Reference surface ocean phosphate concentration
sSi^ref^	6.3 μmol kg^−1^	Reference surface ocean silicate concentration
sDIC^ref^	1,967 μmol kg^−1^	Reference preindustrial surface ocean dissolved inorganic carbon concentration

### 2.3 Biological Pumps and Effects of Ocean Ventilation and Circulation on ∆PCO_2_

We further attribute the interior ocean ∆PCO_2_ variations to the driving biological pump components, that is, the soft‐tissue and carbonate pumps that are associated with the photosynthesis and remineralization of organic matter as well as the precipitation and dissolution of calcium carbonate (CaCO_3_), respectively, and provide an interpretation of the residual term. We make use of a concept that has been developed to separate the interior ocean DIC variations into these contributions (Gruber & Sarmiento, 2002; Sarmiento & Gruber, 2006; Volk & Hoffert, 1985) to estimate their effect on ∆PCO_2_. In order to achieve this goal, we first separately estimate the sDIC, sAlk, sPO_4_, and sSi deviations from their reference values, which are listed in Table 1, defined as ∆X = sX − sX^ref^, for any variable X. For sDIC^ref^, we use the estimated pre‐industrial surface mean value, since the effect of anthropogenic carbon is estimated separately (below and Section 3). Consistent with Gruber and Sarmiento (2002) that used a ${sPO}_{4}^{ref}$ of 0.07 μmol kg^−1^, representing the surface PO_4_ at lower latitudes, we here use a ${sPO}_{4}^{ref}$ of 0.1 μmol kg^−1^. This value is at the upper bound of sPO_4_ in the subtropical gyre regions where nutrient consumption is high and from where organic matter is exported to the deep ocean. This value is substantially smaller than the actual global mean surface value of 0.47 μmol kg^−1^. The elevated mean global surface ocean ${\mathrm{sPO}}_{4}$ largely arises from regions with incomplete nutrient consumption. We also ran sensitivity tests to evaluate the effect of the choice of ${sPO}_{4}^{ref}$ on the deep ocean ∆PCO_2_ using 0.07, 0.1, and 0.47 μmol kg^−1^ (Figure S1 in Supporting Information S1). We found that a ${sPO}_{4}^{ref}$ of 0.47 μmol kg^−1^ leads to a strong positive residual $∆{PCO}_{2}^{res}$ (defined below) with a maximum in the deep northern Pacific (around 1,500 m)—a signal that would rather be expected from the soft‐tissue pump. In contrast, this region should exhibit a local minimum $∆{PCO}_{2}^{res}$ due to the poor ventilation of this region (Holzer et al., 2021). Note that this region may include a large ∆PCO_2_ component that arises from disequilibrium carbon (Eggleston & Galbraith, 2018), which is not included in our estimate of $∆{PCO}_{2}^{res}$ as discussed below. Our sensitivity analysis suggests that a ${sPO}_{4}^{ref}$ of 0.1 μmol kg^−1^ minimizes the effect of a positive $∆{PCO}_{2}^{res}$ in the poorly ventilated deep Pacific, and seems to be a suitable choice. Nevertheless, we note that the separation of ∆PCO_2_ is sensitive to the choice ${sPO}_{4}^{ref}$. Therefore, our results should be interpreted as qualitative rather than quantitative estimates.

We estimate the contributions of the soft‐tissue pump to ∆DIC and ∆Alk by using ∆PO_4_, which results in an estimate of the maximum potential of the soft‐tissue pump in the absence of air‐sea equilibration, that is, the amount of carbon that would be retained in the ocean by the soft‐tissue pump (rsoft) if there was no equilibration at the surface:

(4) $$\begin{array}{l} ∆DIC^{rsoft}=r_{C:P}∆PO_{4}, \end{array}$$

(5) $$\begin{array}{l} ∆Alk^{rsoft}=-r_{N:C}∆DIC^{rsoft}, \end{array}$$

Here, *r*_C:P_ and *r*_N:C_ are the carbon‐to‐phosphate and nitrate‐to‐carbon stoichiometric ratios set to 117/1 and 16/117, respectively (Anderson & Sarmiento, 1994). The latter ratio is applied because a molar increase in seawater nitrate due to for example, the remineralization of organic matter equals the molar decline in Alk (Sarmiento & Gruber, 2006).

We note that this approach makes the assumption that the entire ∆PO_4_ pool in the deep ocean is associated with a corresponding soft‐tissue ∆DIC. In practice, only a fraction of ∆PO_4_ is directly accumulated through remineralization in the deep ocean (so‐called regenerated PO_4_), whereas another fraction of ∆PO_4_ is recirculated through transport and mixing processes after its exposure to the surface ocean (so‐called preformed PO_4_). The latter process is very prominent in the Southern Ocean, where nutrient consumption is incomplete. In order to illustrate this process, we here compute the fraction of the soft‐tissue pump that is associated with directly accumulated carbon (F^accum^) and the fraction that is associated with recirculated carbon (F^recirc^) using the apparent oxygen utilization (AOU) following Williams and Follows (2011) as

(6) $$\begin{array}{l} F^{accum}=\frac{r_{P:O}\left(O_{2}^{sat}\;-\;O_{2}\right)}{∆PO_{4}}, \end{array}$$

and

(7) $$\begin{array}{l} F^{recirc}=1-F^{accum}, \end{array}$$

respectively. Here, the difference between the saturated ($O_{2}^{sat}$) and observed (O_2_) dissolved oxygen concentrations corresponds to AOU, and *r*_P:O_ is the phosphate‐to‐oxygen stoichiometric ratio set to 1/170 (Anderson & Sarmiento, 1994).

While the fraction of ∆PO_4_ that is not consumed by biological production at the surface gets recirculated (F^recirc^), some of the associated DIC is released to the atmosphere through air‐sea equilibration and another part is retained in the ocean as so‐called disequilibrium carbon due to an incomplete air‐sea equilibration at the surface (Eggleston & Galbraith, 2018; Ito & Follows, 2013). Thus, we are here considering the entire history of a water parcel (derived from ∆PO_4_; Sarmiento & Gruber, 2006) and not just the history of the water parcel since it was last at the surface (derived from AOU; Williams & Follows, 2011). While disequilibrium carbon in the existing literature is largely interpreted as an air‐sea gas exchange process, in our study, the retained soft‐tissue pump includes the portion of this disequilibrium carbon that originates from biological sources in the deep ocean, which is not equilibrated at the surface. Moreover, since we attribute all ∆PO_4_ to the soft‐tissue pump, our estimate should be interpreted as the maximum potential of the soft‐tissue pump in the absence of air‐sea equilibration. In contrast to estimating the soft‐tissue pump through AOU (Williams & Follows, 2011), our approach therefore provides the opportunity to estimate the equilibrated DIC at the surface, that is, the fraction of deep ocean carbon that has been released to the atmosphere, through the residual term discussed below.

Subsequently, the contributions of the carbonate pump to the ∆DIC and ∆Alk are estimated as

(8) $$\begin{array}{l} ∆Alk^{carb}=∆Alk-∆Alk^{rsoft}, \end{array}$$

and

(9) $$\begin{array}{l} ∆DIC^{carb}=0.5∆Alk^{carb}, \end{array}$$

respectively.

We proceed to estimate and interpret the pre‐industrial residual DIC term (∆DIC^res^) as:

(10) $$\begin{array}{l} ∆DIC^{res}=sDIC-sDIC^{ref}-∆DIC^{cant}-∆DIC^{rsoft}-∆DIC^{carb}, \end{array}$$

where ∆DIC^cant^ is the anthropogenic DIC estimate (Lauvset et al., 2016; see Section 3). ∆DIC^res^ represents a combination of factors: To first order it includes the part of DIC that has been released to the atmosphere due to incomplete air‐sea equilibration at the surface, that is, ventilation, before the water is subducted again. Since disequilibrium carbon is included in the retained soft‐tissue component, this residual component thus expresses where CO_2_ is actually being gained or lost from the ocean by gas exchange due to equilibration. The residual component also includes effects of the so‐called solubility pump that is associated with DIC changes at the surface due to heating and cooling and subsequent CO_2_ release and uptake, respectively. Moreover, the residual term also includes any errors in our attempt to decompose the DIC pool. In particular, such errors arise from our choice of ${sPO}_{4}^{ref}$ and uncertainties and variations in the stoichiometric ratios. Therefore, our results only provide a qualitative measure.

In contrast to DIC and Alk, PCO_2_ is not a conserved tracer but depends on current carbon chemistry and solubility of the water. Therefore, separating ∆PCO_2_ deviations into its components is not straightforward. Nevertheless, we can quantify the range of values by calculating each contribution, that is, $∆{PCO}_{2}^{rsoft}$, $∆{PCO}_{2}^{carb}$, $∆{PCO}_{2}^{res}$, and $∆{PCO}_{2}^{cant}$ under four scenarios, during which we vary processes that modify the PCO_2_ sensitivity to DIC and Alk, and using p^ref^, S^ref^, and *θ* ^ref^. We run these four scenarios four times for each of the terms above by replacing the terms *x*, y, and *z* in Equations (11), (12), (13), (14) below. In the first run, in order to obtain an estimate for $∆{PCO}_{2}^{rsoft}$, *x* ≡ rsoft, *y* ≡ carb, and *z* ≡ res + cant. In the second run, in order to obtain an estimate for $∆{PCO}_{2}^{carb}$, *x* ≡ carb, *y* ≡ rsoft, *z* ≡ res + cant. In the third run, in order to obtain an estimate for $∆{PCO}_{2}^{res}$, *x* ≡ res, *y* ≡ rsoft, *z* ≡ carb. In the fourth run, in order to obtain an estimate for $∆{PCO}_{2}^{cant}$, *x* ≡ cant, *y* ≡ rsoft, and *z* ≡ carb. For runs where there is no corresponding Alk^x,y,z^, because the process only affects DIC, it is set to zero.

The first scenario for $∆{PCO}_{2}^{x}$ assumes that only this one process influences DIC and Alk deviations from the reference values. As such, $∆{PCO}_{2}^{x}$ is calculated by using sDIC, sAlk, sPO_4_, and sSi fields that are driven by process *x* alone:

(11) $$∆PCO_{2}^{x}=PCO_{2}\left(sDIC^{ref}+∆DIC^{x},sAlk^{ref}+∆Alk^{x},sPO_{4},sSi\right)-PCO_{2}^{ref},$$

where ${PCO}_{2}^{ref}$ is the ocean PCO_2_ calculated using all reference values (Table 1). The second and third scenarios assume that one of the two other processes *y* or *z* influencing DIC and Alk has occurred prior to process *x* to determine the PCO_2_ sensitivity to DIC and Alk:

(12) $$\begin{array}{l} ∆PCO_{2}^{x}=\\ PCO_{2}\left(sDIC^{ref}+∆DIC^{y}+∆DIC^{x},\;sAlk^{ref}+∆Alk^{y}+∆Alk^{x},sPO_{4},sSi\right)-\\ PCO_{2}\left(sDIC^{ref}+∆DIC^{y},\;sAlk^{ref}+∆Alk^{y},sPO_{4}^{ref},sSi^{ref}\right), \end{array}$$

(13) $$\begin{array}{l} ∆PCO_{2}^{x}=\\ PCO_{2}\left(sDIC^{ref}+∆DIC^{z}+∆DIC^{x},sAlk^{ref}+∆Alk^{z}+∆Alk^{x},sPO_{4},sSi\right)-\\ PCO_{2}\left(sDIC^{ref}+∆DIC^{z},sAlk^{ref}+∆Alk^{z},sPO_{4}^{ref},sSi^{ref}\right). \end{array}$$

The last scenario is that process *x* occurs after all the other processes. Therefore, we have

(14) $$\begin{array}{l} ∆PCO_{2}^{x}=PCO_{2}\left(sDIC,sAlk,sPO_{4},sSi\right)-\\ PCO_{2}\left(sDIC-∆DIC^{x},sAlk-∆Alk^{x},sPO_{4}^{ref},sSi^{ref}\right). \end{array}$$

We then use the mean of the four scenarios (Equations (11), (12), (13), (14)) as an estimate for the contribution of the $∆{PCO}_{2}^{x}$ to the interior ocean ∆PCO_2_ distribution.

### 2.4 Impact of Carbon Chemistry on the Vertical ∆PCO_2_ Distribution

To estimate how the seawater carbon chemistry drives the vertical ∆PCO_2_ distribution, we separately estimate the departure of ∆PCO_2_ due to deviations of DIC and Alk from the respective reference values (Table 1). The increasing DIC with depth is the primary source of the elevated ∆PCO_2_ in the deeper layers of the ocean, which is then modified by Alk. Therefore, we first estimate PCO_2_ variations due to DIC alone ($∆{PCO}_{2}^{DIC}$):

(15) $$\begin{array}{l} ∆PCO_{2}^{DIC}=PCO_{2}^{DIC}-PCO_{2}^{ref}=\\ PCO_{2}\left(sDIC,sAlk^{ref},\theta^{ref},S^{ref},sPO_{4},sSi\right)-PCO_{2}^{ref}. \end{array}$$

We then estimate the impact of Alk variations ($∆{PCO}_{2}^{Alk}$) as

(16) $$\begin{array}{l} ∆PCO_{2}^{Alk}=PCO_{2}^{DIC,Alk}-PCO_{2}^{DIC}-PCO_{2}^{ref} \end{array}$$

where ${PCO}_{2}^{DIC,Alk}$ is defined in Section 2.2. Moreover, we define $∆{PCO}_{2}^{DIC,Alk}={\;PCO}_{2}^{DIC,Alk}-p{CO}_{2}^{atm}$.

## 3 Data

Our conclusions are drawn from ship‐based measurements and measurements by profiling floats. We use physical and biogeochemical data from the gridded Global Ocean Data Analysis Project version 2 (GLODAPv2; Key et al., 2004, 2015; Lauvset et al., 2016; Olsen et al., 2016) climatology, which are provided at a 1° by 1° spatial resolution. GLODAPv2 is based on shipboard measurements from 724 hydrographic cruises from 1972 to 2013. Estimates of anthropogenic DIC provided in the mapped GLODAPv2 data (Lauvset et al., 2016) were used to assess the anthropogenic contribution to DIC. The GLODAPv2 DIC and ∆DIC^cant^ estimates are referenced to the year 2002. In addition, we use biogeochemical profiling float data from a broad region in the Southern Ocean (Johnson et al., 2021), which were deployed as part of the Southern Ocean Carbon and Climate Observations and Modeling (SOCCOM) project (Johnson et al., 2017). Here, we use a total of 8,332 profiles from 174 floats with measurements of high‐quality pH (Johnson et al., 2016; Williams et al., 2017), NO_3_, and estimated Alk (Carter et al., 2018) to estimate PCO_2_.

Surface CO_2_ flux data stems from two neural‐network estimates; one is weighted toward biogeochemical Argo float data (Bushinsky et al., 2019; Landschützer et al., 2019) and one uses only shipboard observations from the SOCAT database (Landschützer et al., 2016, 2020). A third product is a climatology based on shipboard observations from the CDIAC database (Takahashi et al., 2009). Climatological frontal positions stem from Orsi et al. (1995) and the sea‐ice edge position is the annual mean 1% sea‐ice concentration derived from the NSIDC Climate Data Record (1979–2018; Meier et al., 2017; Peng et al., 2013).

## 4 Results

### 4.1 Spatial Patterns of CO_2_ Release Linked to Subsurface ∆PCO_2_ Maximum

**Figure 2:**
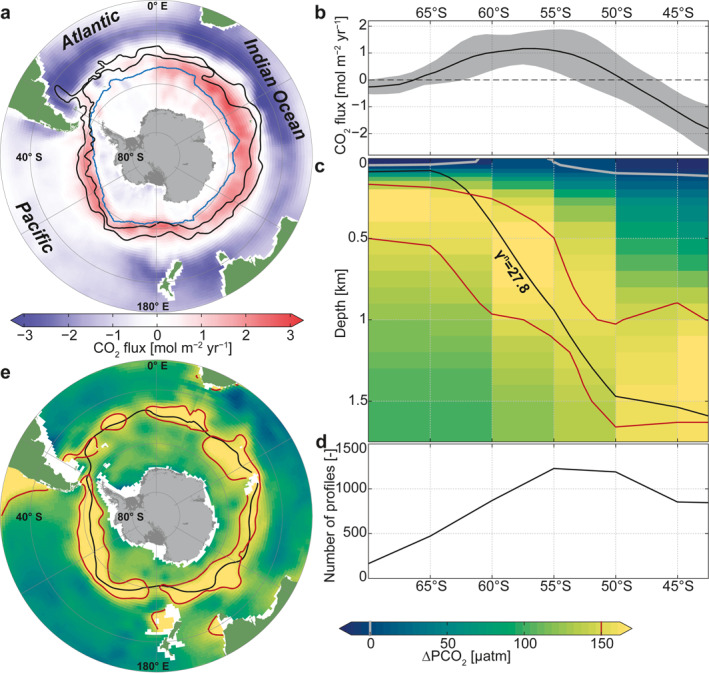
Southern Ocean CO_2_ release and its subsurface source. (a) Annual mean surface CO_2_ flux from a neural‐network estimate that is weighted with biogeochemical Argo float data (Bushinsky et al., 2019; Landschützer et al., 2019). Positive values (red) indicate CO_2_ degassing from the ocean to the atmosphere. Black contours denote the mean position of the Subantarctic and Polar Fronts (Orsi et al., 1995). The light blue contour line represents the annual mean sea‐ice edge (1% mean sea‐ice concentration). (b) Zonally averaged annual mean surface CO_2_ flux (as in panel (a)). Gray shading denotes ±1 standard deviation in the zonal direction. (c) Zonally averaged annual‐mean potential *p*CO_2_ excess with respect to current atmospheric levels (400 μatm; ∆PCO_2_) from profiling floats (in 5° latitudinal bins). (d) Number of float profiles per 5° latitudinal bins. (e) ∆PCO_2_ at 800 m from gridded GLODAPv2. In panels (c and e), the 27.8 kg m^−3^ isoneutral surface is shown in black, and the 150 and 0 μatm ∆PCO_2_ isolines in red and gray, respectively.

The float‐weighted annual mean surface CO_2_ flux estimate (Bushinsky et al., 2019; Landschützer et al., 2019) reveals a distinct ring feature (Figures 2a and 2b), with strong CO_2_ release occurring within the ACC, roughly between the Subantarctic Front and the winter‐time sea‐ice edge. The highest outgassing occurs in the Pacific and Indian Ocean sectors, regionally exceeding 3 mol m^−2^ yr^−1^ (positive values are a CO_2_ flux from the ocean to the atmosphere; see also Prend et al. (2022)). In these regions, intensive CO_2_ release is observed in the fall and winter, followed by weaker, yet non‐negligible, outgassing fluxes in the remaining seasons (Bushinsky et al., 2019; Gray et al., 2018). North of the ACC, CO_2_ fluxes are characterized by a broad region of CO_2_ uptake. The highest annual uptake, below −3 mol m^−2^ yr^−1^, is found in the Argentine Basin in the Atlantic. In the seasonally ice‐covered region, annual mean surface CO_2_ fluxes are generally small. In summary, the largest CO_2_ release occurs roughly between the sea‐ice edge and the Subantarctic Front during wintertime, a region that is dominated by a wind‐driven divergence at the surface and deep mixing in winter (Bushinsky et al., 2019; Gray et al., 2018; Prend et al., 2022) and during strong storms (Nicholson et al., 2022).

**Figure 3:**
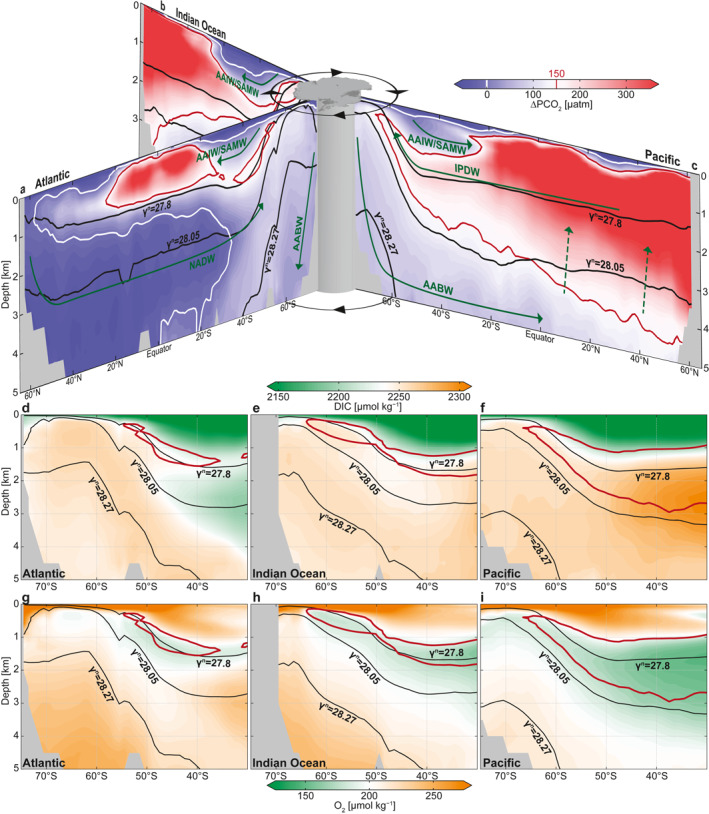
Potential *p*CO_2_ excess above current atmospheric levels (400 μatm; ∆PCO_2_) and dissolved inorganic carbon (DIC) in the ocean interior from gridded GLODAPv2. Meridional sections of zonally averaged ∆PCO_2_ in the Atlantic (a), Indian (b), and Pacific (c) Oceans, overlaid with a schematic representation of the subsurface water pathways (white arrows) and the Antarctic Circumpolar Current (black arrows). Zonally averaged DIC in the Atlantic (d), Indian (e), and Pacific (f) sectors of the Southern Ocean (south of 30°S). Zonally averaged dissolved oxygen (O_2_) in the Atlantic (g), Indian Ocean (h), and Pacific (i) sectors of the Southern Ocean (south of 30°S). Isoneutral surfaces are shown in black (*γ* ^N^ = 27.8 and 28.05 kg m^−3^ characterizing Indo‐Pacific Deep Water [IPDW] and North Atlantic Deep Water [NADW], respectively), and the 150 and 0 μatm ∆PCO_2_ isolines in red and white, respectively.

The ring pattern of outgassing evident from the surface CO_2_ fluxes aligns with the region of upward sloping isopycnal surfaces in the Southern Ocean subsurface between about 55° and 65°S (Figures 2c and 2d). In this region, the zonal mean ∆PCO_2_ from the float profiles reveals a signature of high‐∆PCO_2_ deep water that upwells along isopycnals. The vertical ∆PCO_2_ maximum appears along the 27.8 kg m^−3^ neutral density surface, which shoals toward the south within the ACC; this is coincident with the core of the oxygen minimum layer that is characteristic of, and defines, IPDW (Talley, 2013; see also Figures 3g–3i). The sign of the surface CO_2_ fluxes (Figure 2b) closely tracks this shoaling of the subsurface ∆PCO_2_ maximum (Figure 2c), switching from CO_2_ uptake north of 50°S to CO_2_ outgassing between about 50°S and 65°S (Figure 2b). Maximum outgassing, between 55° and 60°S, encompasses the latitude range of positive ∆PCO_2_ at the surface (gray contour lines in Figure 2c). This picture of highest surface outgassing in the region where high‐∆PCO_2_ waters upwell, as derived from the profiling floats, is corroborated by a ring pattern in the ∆PCO_2_ constructed from gridded shipboard observations (Section 3) at 800 m depth (Figure 2e). It reflects the high annual‐mean surface CO_2_ fluxes, with highest values occurring between the Polar and Subantarctic Fronts. The core of the subsurface high‐∆PCO_2_ ring coincides with the outcropping of the 27.8 kg m^−3^ neutral density surface (black in Figures 2c and 2e). Both the surface CO_2_ fluxes and the subsurface ∆PCO_2_ at 800 m decrease further south of the outcropping 27.8 kg m^−3^ neutral density surface. Therefore, the reduced CO_2_ outgassing south of its maximum is in part related to a decrease of the subsurface ∆PCO_2_ south of 60°S. In addition, the inhibition of gas exchange by sea‐ice cover, a potentially high biological carbon export efficiency (Arteaga et al., 2018), reduced mixing (Wilson et al., 2019), and cold surface temperatures could further limit the mixed‐layer ∆PCO_2_ and thereby surface CO_2_ fluxes in this seasonally ice‐covered region. In summary, the outcropping of the isopycnal surfaces in the Southern Ocean acts to project the vertical ∆PCO_2_ structure with its maximum on the 27.8 kg m^−3^ neutral density surface onto a horizontal plane, resulting in the meridional ∆PCO_2_ maximum between the sea‐ice edge and Subantarctic Front.

### 4.2 Sources of the Subsurface ∆PCO_2_ Maximum

Where does the high subsurface ∆PCO_2_ water in the Southern Ocean come from? In order to address this question, in the following, we base our analysis on ∆PCO_2_ derived from a global data set (GLODAPv2; Lauvset et al., 2016; see Section 3) as displayed in Figure 3. Note that this data has several advantages, such as higher accuracy ship‐board observations compared to the float data, actual alkalinity observations, and being a gridded product. However, major differences occur at the Southern Ocean surface. Here, the float data suggest an outcrop of the zero ∆PCO_2_ contour (gray in Figure 2c), whereas the the zero ∆PCO_2_ contour from ship‐data (white in Figures 3a–3c) does not outcrop. This difference most likely occurs from the lack of wintertime observations in GLODAPv2. In this study, we are mostly interested in the subsurface ∆PCO_2_, where both data sets largely agree.

We now assume that, to a first order, water masses spread along isoneutral density surfaces (denoted as *γ* ^N^)—an assumption that has been widely used to identify the deep water circulation pathways (Jackett & McDougall, 1997). The ∆PCO_2_ maximum follows the 27.8 kg m^−3^ isoneutral surface, which characterizes the IPDW core as identified by the vertical oxygen minimum (Talley, 2013, Figure 3); hence the vertical oxygen and ∆PCO_2_ extrema appear to have the same biological and physical forcing. The ∆PCO_2_ maximum can be traced back from the Southern Ocean subsurface to its source region in the deep northern Indian and Pacific basins (Figures 3a–3c). Here, the ocean is pervaded by PCO_2_‐rich waters, with ∆PCO_2_ much larger than 150 μatm extending from the thermocline to about 4 km depth (red contour line in Figures 3b and 3c).

The highest ∆PCO_2_ waters, indicated by a ∆PCO_2_ larger than 800 μatm, are located in the North Pacific between 50° and 60°N and between 500 and 1,500 m, where the oldest subsurface waters occur in the global ocean, supplied by slow vertical diffusion from below (dashed white lines in Figure 3c; Holzer et al., 2021; Talley, 2013). These waters with high ∆PCO_2_ spread southward and outcrop at the sea surface in the Southern Ocean (Gent & McWilliams, 1990; Holzer et al., 2021; Talley, 2013). They enter the Southern Ocean as a layer of elevated ∆PCO_2_ that extends southward and upward across 50°S, where the zonal mean PCO_2_ exceeds atmospheric levels by about 175 ± 32 μatm (averaged between 27.7 and 27.9 kg m^−3^; Table 2). Both advective and diffusive eddy transport can play an important role in pulling these high ∆PCO_2_ signals to the Southern Ocean subsurface (Dufour et al., 2015; Tamsitt et al., 2017), with the final step being entrainment into the surface mixed layer (Prend et al., 2022). This chimney of elevated ∆PCO_2_ is narrowly constrained along the IPDW core (27.8 kg m^−3^ isoneutral surface; Figure 3c). As these waters reach the Southern Ocean, the ∆PCO_2_ signal is strongly attenuated as a result of diapycnal mixing with ambient low ∆PCO_2_ waters, yet its magnitude is still strongly elevated and forms a clear local vertical maximum in all three ocean basins. South of the Polar Front, where the 27.8 kg m^−3^ isoneutral surface outcrops, the high ∆PCO_2_ signals originated from the IPDW are circumpolarly distributed, again mirroring the oxygen minimum layer (Figures 3g–3i; Talley, 2013), and control the subsurface ∆PCO_2_ around Antarctica.

**Table 2:** Deep Ocean ∆PCO_2_ (in µatm), Reference Values (Ref), and the Estimated Sources From Temperature (T) and Salinity (S), and Dissolved Inorganic Carbon (DIC) and Alkalinity (Alk) Variations

Water mass		IPDW	LCDW/NADW	AABW	LCDW/NADW–IPDW	AABW–IPDW
Range		27.7 kg m^−3^–27.9 kg m^−3^	27.9 kg m^−3^–28.2 kg m^−3^	>28.2 kg m^−3^		
∆PCO_2_		175 ± 32	104 ± 33	77 ± 16	−70 ± 46	−97 ± 36
Ref	$p{CO}_{2}^{atm}$	−400	−400	−400	0	0
	${PCO}_{2}^{ref}$	+131	+131	+131	0	0
T, S	$∆{PCO}_{2}^{dil}$	−1 ± 1	0 ± 0	0 ± 0	+2 ± 1	+1 ± 1
	$∆{PCO}_{2}^{sol}$	−4 ± 7	−23 ± 11	−53 ± 8	−19 ± 13	−48 ± 10
DIC, Alk	$∆{PCO}_{2}^{rsoft}$	+595 ± 59	+506 ± 59	+530 ± 22	−89 ± 84	−65 ± 63
	$∆{PCO}_{2}^{carb}$	−112 ± 22	−104 ± 24	−114 ± 6	+8 ± 32	−2 ± 23
	$∆{PCO}_{2}^{cant}$	+39 ± 17	+18 ± 9	+25 ± 9	−21 ± 19	−13 ± 19
	$∆{PCO}_{2}^{res}$	−78 ± 29	−25 ± 19	−46 ± 16	+53 ± 34	+33 ± 33
	$∆{PCO}_{2}^{dic}$	+699 ± 92	+676 ± 105	+707 ± 31	−23 ± 140	+8 ± 98
	$∆{PCO}_{2}^{alk}$	−253 ± 70	−283 ± 83	−312 ± 24	−30 ± 108	−59 ± 74

*Note.* DIC and Alk variations are also split into contributions by the retained soft‐tissue (rsoft) and carbonate (carb) pumps, as well as the anthropogenic and residual contributions. Values are averaged for different water masses (columns 2, 3, and 4) and differences between water masses and IPDW (columns 5 and 6). All values are averaged between 45° and 55°S. Note that the components do not add up to zero due to non‐linearities in the PCO_2_ estimation and should be understood as a qualitative measure. Uncertainties are reflected by the respective standard deviation.

In contrast to the maximum ∆PCO_2_ found in IPDW, the lowest value of deep‐ocean ∆PCO_2_, of less than −50 μatm, is located in the northern Atlantic, where NADW is formed from surface sources, and hence has higher oxygen and lower ∆PCO_2_ than IPDW (Figures 3a and 3g). The NADW core in the Southern Ocean has been identified by its salinity maximum, which roughly follows the 28.05 kg m^−3^ isoneutral surface (Talley, 2013). While the ∆PCO_2_ of NADW increases continuously due to diapycnal mixing and biological processes as the NADW moves southward, much less CO_2_ outgassing would be expected if NADW were the primary water mass being ventilated in the Southern Ocean. In the Southern Ocean, NADW mixes with higher ∆PCO_2_ LCDW from the Indo‐Pacific (Figure 3c). Between 45° and 55°S, the circumpolar average of LCDW/NADW has a ∆PCO_2_ that is about 70 ± 46 μatm lower than the ∆PCO_2_ of IPDW (Table 2). This LCDW/NADW mixture then upwells primarily in the region covered by the seasonal sea ice (Talley, 2013), where the local environment further hinders the air‐sea CO_2_ exchange. In conclusion, our analysis shows that the characteristic pattern of a high ΔPCO_2_ ring below the mixed layer, which causes the Southern Ocean CO_2_ outgassing, is induced by the southward and upward transport of IPDW through a narrow and relatively light isoneutral density band and cannot be explained by the upwelling of LCDW, such as NADW.

### 4.3 Drivers of High ∆PCO_2_ in Indo‐Pacific Deep Water

**Figure 4:**
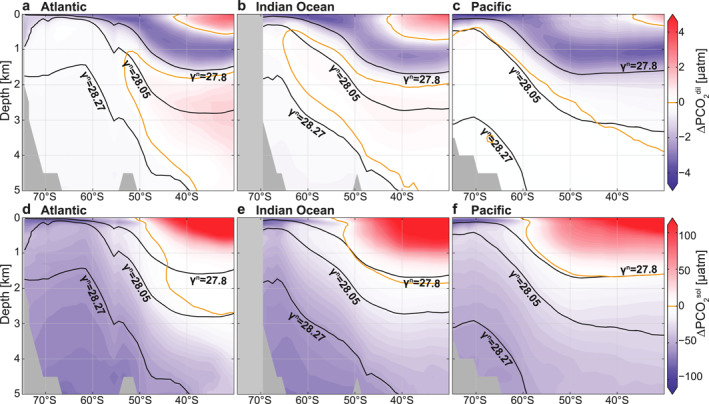
Temperature and salinity effects on the subsurface ∆PCO_2_ structure. (a–c) Dilution effects ($∆{PCO}_{2}^{dil}$) relative to a salinity of 34.7. (d–f) Solubility effects ($∆{PCO}_{2}^{sol}$) with respect to a reference temperature and salinity of 2.5°C and 34.7°C, respectively. Panels show the Atlantic (left), Indian (middle), and Pacific (right) sectors of the Southern Ocean. Isoneutral surfaces are shown in black, and the 0 μatm ∆PCO_2_ isoline in orange.

**Figure 5:**
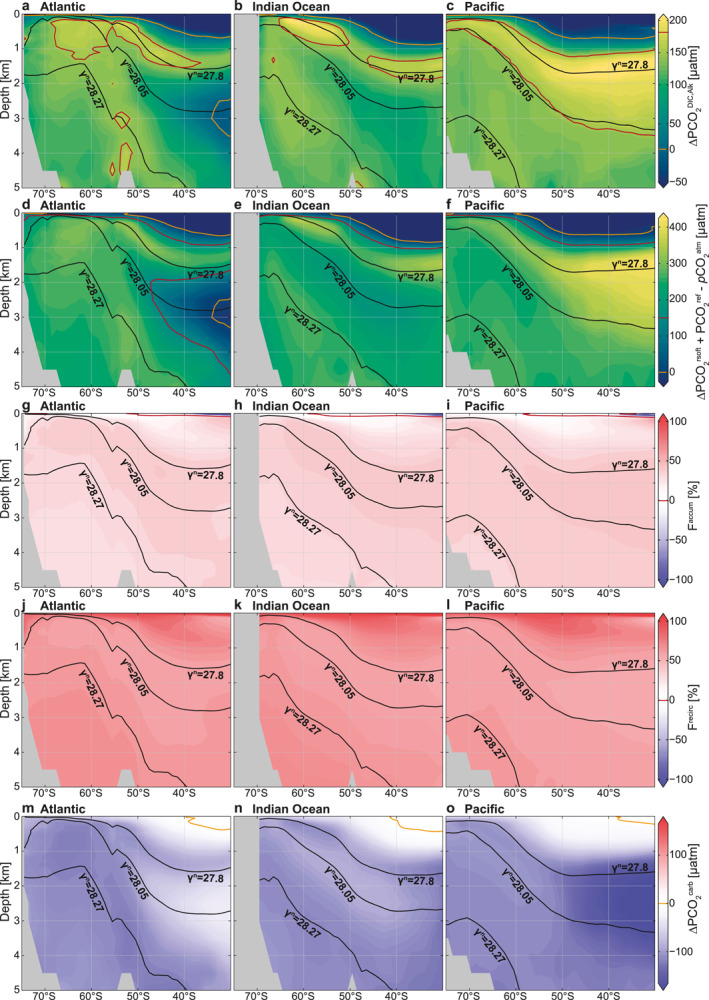
Biological drivers of the subsurface ∆PCO_2_ structure. (a–c) ∆PCO_2_ structure due to interior ocean dissolved inorganic carbon (DIC) and Alk variations alone ($∆{PCO}_{2}^{DIC,Alk}$), after removing dilution and solubility effects. (d–f) Expected ∆PCO_2_ driven by the retained soft‐tissue pump ($∆{PCO}_{2}^{rsoft}$) due to photosynthesis and remineralization of organic matter plus ${PCO}_{2}^{ref}$ (131 μatm) minus $p{CO}_{2}^{atm}$ (400 μatm). (g–i) Fraction of the retained soft‐tissue pump associated with accumulated remineralized carbon in the deep ocean (F^accum^). (j–l) Fraction of the retained soft‐tissue pump recirculated remineralized carbon (F^recirc^). (m–o) Carbonate pump ($∆{PCO}_{2}^{carb}$) due to precipitation and dissolution of calcium carbonate. Panels show the Atlantic (left), Indian (middle), and Pacific (right) sectors of the Southern Ocean. Isoneutral surfaces are shown in black, and the 150 and 0 μatm ∆PCO_2_ isolines in red and orange, respectively.

**Figure 6:**
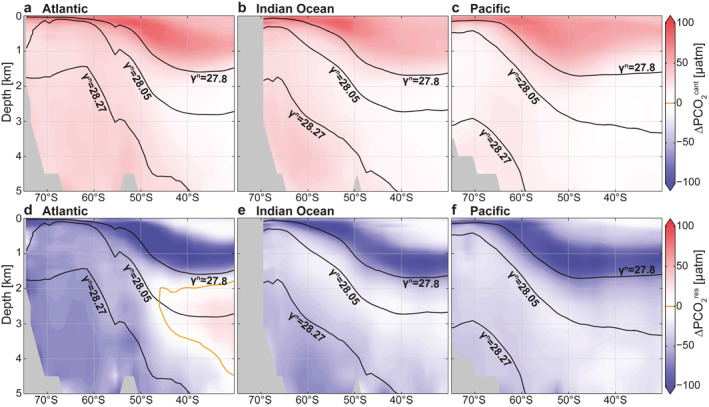
Ventilation effects on the subsurface ∆PCO_2_ structure. (a–c) Influence of anthropogenic carbon uptake ($∆{PCO}_{2}^{cant}$). (d–f) Influence of air‐sea gas exchange due to equilibration and other residual effects ($∆{PCO}_{2}^{res}$). Panels show the Atlantic (left), Indian (middle), and Pacific (right) sectors of the Southern Ocean. Isoneutral surfaces are shown in black, and the 0 μatm ∆PCO_2_ isoline in orange.

**Figure 7:**
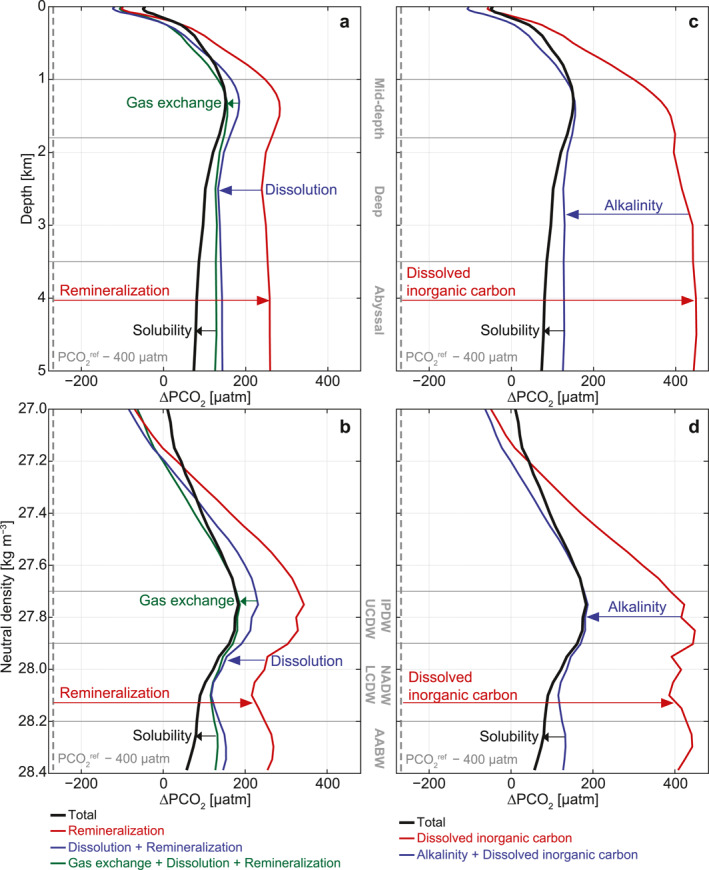
Drivers of the vertical ∆PCO_2_ profile between 45° and 55°S. Decomposition of ∆PCO_2_ profiles into biological and physical processes in (a) depth and (b) neutral density coordinates. Decomposition of ∆PCO_2_ profiles into chemical (dissolved inorganic carbon, Alk) and physical components in (c) depth and (d) neutral density coordinates. Note that in this figure, for simplicity, gas exchange is the combination of anthropogenic and natural components and dilution effects are neglected.

Why is ∆PCO_2_ particularly elevated along the circulation pathway of IPDW? A larger CO_2_ outgassing potential in deep waters has generally been linked to a higher DIC content. However, IPDW has no clear vertical DIC maximum in the Southern Ocean (Figures 3d–3f). Instead, vertically, the DIC peaks are located at a much greater depth and higher density than those of ∆PCO_2_. For example, DIC maxima in the Southern Ocean occur on isoneutral surfaces that are associated with the Antarctic Bottom Water (AABW; *γ* ^N^ ∼28.27 kg m^−3^; Orsi et al., 1999) in the Atlantic and Indian Ocean (Figures 3d and 3e), and on isoneutral density surfaces of about 28.0 kg m^−3^ in the Pacific north of 50°S (Figure 3f). Since the IPDW ∆PCO_2_ maximum cannot simply be explained by its carbon content (DIC), we here consider the different temperature and salinity effects (dilution and solubility; Section 4.3.1; Figure 4), and DIC and Alk variations arising from biological and ventilation processes (Section 4.3.2; Figures 5 and 6), as well as the carbonate chemistry (Section 4.3.3; Figure 7) that determine the interior Southern Ocean PCO_2_ structure (see Section 2 for methods). For this purpose, we evaluate these contributions on depth levels (Figures 4, 5, 6, 7a and 7b) and on neutral density surfaces that represent the corresponding water masses (Figures 7c and 7d, Table 2). We vertically separate the subsurface ocean into three neutral density layers: IPDW (27.7–27.9 kg m^−3^), LCDW/NADW (27.9–28.2 kg m^−3^), and AABW (>28.2 kg m^−3^) (Figure 7, Table 2).

#### 4.3.1 Temperature and Salinity Effects on Interior Ocean ∆PCO_2_ Structure

Physical effects of interior ocean temperature and salinity variations on ∆PCO_2_ include the dilution of ocean tracers by surface freshwater fluxes, and changes in CO_2_ solubility (Section 2.2). Dilution effects (∆${PCO}_{2}^{dil}$; Figures 4a–4c, Table 2) are generally small (<5 μatm). Compared to our chosen reference salinity of 34.7, the LCDW/NADW layer (*γ* ^N^ ∼ 28.05 kg m^−3^) and the subtropical surface waters have an elevated $∆{PCO}_{2}^{dil}$ due to evaporation effects on those waters. Antarctic surface waters, Antarctic Intermediate Water (AAIW), and Subantarctic Mode Water (SAMW) experience a reduced $∆{PCO}_{2}^{dil}$ due to a net freshwater input at the Southern Ocean surface. However, this pattern cannot explain the interior ocean ∆PCO_2_ structure and the order of magnitude of $∆{PCO}_{2}^{dil}$ is negligible.

Solubility effects ($∆{PCO}_{2}^{sol}$; Figures 4d–4f), which are mostly associated with the interior Southern Ocean temperature distribution and to a lesser degree with the salinity distribution, are on the order of about 20–100 μatm. They become very large in the much warmer subtropical thermocline waters. Overall, the colder temperatures in the deepest layers, in particular AABW, as well as the high‐latitude Antarctic surface waters, reduce ∆PCO_2_ and enhance the ability of these waters to hold CO_2_ or reduce their outgassing potential. Thus, solubility effects have a substantial influence on the interior Southern Ocean ∆PCO_2_ structure and act to contribute to the observed vertical ∆PCO_2_ decrease, especially in the deepest layers (Figures 7a and 7b, Table 2), with the most negative values occurring in the deep southern Atlantic (Figure 4d). However, vertical $∆{PCO}_{2}^{sol}$ gradients around the ∆PCO_2_ maximum (27.8 kg m^−3^) are weaker than in the abyssal ocean, suggesting that other processes control the ∆PCO_2_ decrease with depth, especially between the IPDW and LCDW/NADW layers. In summary, dilution effects are small and solubility effects on ∆PCO_2_, arising from interior ocean temperature variations, enforce the IPDW ∆PCO_2_ maximum (Table 2).

#### 4.3.2 Biological and Ventilation Effects on Interior Ocean ∆PCO_2_ Structure

In order to better understand the biological and ventilation contribution to the subsurface ∆PCO_2_ structure in the Southern Ocean, we compare the contributions of the retained soft‐tissue pump ($∆{PCO}_{2}^{rsoft}$), carbonate pump ($∆{PCO}_{2}^{carb}$), and residual ($∆{PCO}_{2}^{res}$) to ∆PCO_2_ variations that arise from variations in sDIC and sAlk alone ($∆{PCO}_{2}^{DIC,Alk}$; Figures 5a–5c), that is, for fixed reference values of temperature, salinity, and pressure (Section 2). It should be noted that while these reference values affect the actual value of ∆PCO_2_, they do not affect the relative partitioning between the factors driving $∆{PCO}_{2}^{DIC,Alk}$. We also note that $∆{PCO}_{2}^{DIC,Alk}$ approximately equals the sum of ${PCO}_{2}^{ref}$, $∆{PCO}_{2}^{rsoft}$, $∆{PCO}_{2}^{carb}$, $∆{PCO}_{2}^{res}$, and $∆{PCO}_{2}^{cant}$ minus $p{CO}_{2}^{atm}$ (Section 2). These contributions are discussed in this section.

The retained soft‐tissue pump ($∆{PCO}_{2}^{rsoft}$) is the most efficient mechanism to increase the deep ocean's ∆PCO_2_ through the accumulation and subsequent recirculation of remineralized carbon (DIC; Figure 7, Table 2). The contribution of this mechanism to ∆PCO_2_ explains most of the interior ocean structure of $∆{PCO}_{2}^{DIC,Alk}$ (Figures 5a–5f), as well as the occurrence of very high $∆{PCO}_{2}^{DIC,Alk}$ in IPDW, which is especially prominent in the Pacific (Figure 5f). High values of $∆{PCO}_{2}^{rsoft}$ in the rather shallow IPDW (Table 2), as compared to the deeper LCDW/NADW (Table 2), partially occur because IPDW accumulates respired carbon over long timescales (DeVries & Primeau, 2011), which is related to the long route that newly ventilated AABW takes through the Indian and Pacific, upwelling diffusively to produce IPDW, which then returns to the Southern Ocean surface (Holzer et al., 2021; Talley, 2013). In addition to the difference in accumulation time, IPDW receives more organic carbon from remineralization as a result of its rather shallow depth in the water column (shallower than 1,500 m) compared to LCDW/NADW and AABW (Kwon et al., 2009; Martin et al., 1987). The organic matter received by IPDW could be about twice as large as in the layers below when using a vertical organic carbon remineralization curve that follows a classic power‐law relationship (Martin et al., 1987) and ignoring possible spatial differences in the production of organic matter at the surface. A secondary peak in both $∆{PCO}_{2}^{DIC,Alk}$ and $∆{PCO}_{2}^{rsoft}$ occurs around the 28.27 kg m^−3^ isoneutral surface, which marks the upper bound of AABW (Orsi et al., 1999), in particular in the Weddell Sea (Atlantic south of 50°S; Figures 5a and 5d). This secondary $∆{PCO}_{2}^{DIC,Alk}$ peak arises from local production and remineralization within the Weddell Gyre (MacGilchrist et al., 2019). A local minimum is found in the core of the relatively young NADW around the 28.05 kg m^−3^ isoneutral surface in the Atlantic (Figure 5d; DeVries & Primeau, 2011), which disappears toward the south as this water masses mixes with other waters in the Southern Ocean. In conclusion, $∆{PCO}_{2}^{rsoft}$ dominates the vertical ∆PCO_2_ decline below IPDW, which also explains the colocation of the vertical ∆PCO_2_ maximum and the dissolved oxygen minimum in the deep ocean (Section 4.2), because both quantities are dominated by the soft‐tissue pump.

Our estimate of $∆{PCO}_{2}^{rsoft}$ should be interpreted as the maximum potential of the retained soft‐tissue pump in the absence of air‐sea equilibration at the surface, since it is estimated from the total ∆PO_4_. In practice, when the upwelling water masses reach the surface of the Southern Ocean, PO_4_ that is not utilized by biological production becomes recirculated into the ocean interior with the subduction of AAIW, SAMW, and AABW. In the absence of air‐sea equilibration this recirculated PO_4_ would also be associated with recirculated biogenic DIC^rsoft^. However, a partial air‐sea equilibration of DIC occurs at the surface. The equilibrated DIC portion is part of our residual estimate ($∆{PCO}_{2}^{res}$), further discussed below, and the portion of soft‐tissue carbon associated with the incomplete equilibration (so‐called disequilibrium carbon; Eggleston & Galbraith, 2018; Ito & Follows, 2013) is part of our $∆{PCO}_{2}^{rsoft}$ and is thus interpreted as biogenic carbon in our context (see Section 2.3). Further insight into the contribution of the directly accumulated fraction (F^accum^) and the recirculated fraction (F^recirc^) to $∆{PCO}_{2}^{rsoft}$ is gained by partitioning them using AOU (Section 2.3; Williams & Follows, 2011). These two contributions (Figures 5g–5l) highlight the important potential of the ocean circulation to recirculate carbon in the ocean in the absence of air‐sea equilibration since F^recirc^ dominates $∆{PCO}_{2}^{rsoft}$ in AABW, AAIW, and SAMW. In the IPDW core, between the 27.7 and 27.9 kg m^−3^ isoneutral surfaces, F^accum^ becomes as large as 39% but still remains smaller than F^recirc^. This overall large contribution of F^recirc^ implies that any long‐term changes in the surface equilibration due to, for example, changes in the surface residence time, sea ice cover, or solubility of these waters could alter the carbon storage in the deep ocean. In addition, a shift in the occupied deep ocean volume from NADW (low recirculated soft‐tissue carbon) to AABW (high recirculated soft‐tissue carbon), as implied for glacial times (Curry & Oppo, 2005), could substantially impact the total CO_2_ balance between the ocean and the atmosphere due to an increased recirculation of soft‐tissue carbon in the deep ocean (Eggleston & Galbraith, 2018; Skinner, 2009).

The magnitude of $∆{PCO}_{2}^{rsoft}$ (Figures 5d–5f) is substantially larger than $∆{PCO}_{2}^{DIC,Alk}$ (Figures 5a–5c), because the carbonate pump ($∆{PCO}_{2}^{carb}$; Figures 5m–5o) and residual terms ($∆{PCO}_{2}^{res}$; Figures 5g–5i) counter the retained soft‐tissue pump effect on $∆{PCO}_{2}^{DIC,Alk}$. The negative $∆{PCO}_{2}^{carb}$ in the deep ocean arises from the dissolution of mineral carbonates, which increases alkalinity twice as much as it increases DIC. The most negative values of $∆{PCO}_{2}^{carb}$ are located in the deep Pacific (Figure 5o), where dissolution of mineral carbonate is enhanced by the metabolic CO_2_ release that drives the deep water to be undersaturated with respect to calcite (Broecker & Peng, 1987; Jiang et al., 2015; Kwon et al., 2011; Sarmiento et al., 1988). As the dissolution process strongly depends on pressure and preferentially happens at greater depth compared to organic matter remineralization (Broecker & Peng, 1987; Kwon et al., 2011; Sarmiento et al., 1988), the $∆{PCO}_{2}^{carb}$ deficit appears to peak at greater depth than the $∆{PCO}_{2}^{rsoft}$ maximum in the Pacific (Figure 5o). However, due to compensatory effects between the Atlantic and the Pacific basins (Figures 5m and 5o), the $∆{PCO}_{2}^{carb}$ contribution to the overall zonal mean vertical ∆PCO_2_ decrease with depth is small (Figure 7a; between 45° and 55°S) and even of opposite sign when averaged over neutral density layers (Table 2). Therefore, the overall effects of $∆{PCO}_{2}^{carb}$ in setting the vertical ∆PCO_2_ maximum in IPDW are small, but are critical to understanding the differences between the vertical ∆PCO_2_ and DIC profiles, as we show in Section 4.3.3.

The effect of anthropogenic carbon on ∆PCO_2_ ($∆{PCO}_{2}^{cant}$; Figures 6a–6c) is estimated based on GLODAPv2 (Section 2.3). $∆{PCO}_{2}^{cant}$ is elevated in the AAIW, SAMW, and subtropical surface waters and also has some smaller positive values in the AABW, due to an uptake of anthropogenic carbon by these waters before they are subducted (Gruber et al., 2019). However, a substantial amount of $∆{PCO}_{2}^{cant}$ is also found in IPDW, and the decline of $∆{PCO}_{2}^{cant}$ with depth contributes to the overall vertical ∆PCO_2_ decline below IPDW (Table 2). Such an invasion of $∆{PCO}_{2}^{cant}$ to the IPDW layer could arise from along‐isopycnal stirring by mesoscale eddies, connecting the surface outcrop of the isopycnal with the older, upwelling deep waters (Abernathey & Ferreira, 2015). Overall, anthropogenic carbon reinforces the IPDW ∆PCO_2_ maximum (Table 2).

$∆{PCO}_{2}^{res}$ (Figures 6d–6f) includes all residual effects of unexplained subsurface DIC variations. These effects include carbon release at the ocean surface due to air‐sea equilibration which acts to lower the subsurface $∆{PCO}_{2}^{res}$ because the recirculated DIC pool becomes depleted by carbon outgassing to the atmosphere compared to the recirculated PO_4_ pool (see discussion above). Therefore, negative $∆{PCO}_{2}^{res}$ values reflect CO_2_ that was released from the ocean due to ventilation processes. The most negative values of $∆{PCO}_{2}^{res}$ are associated with AAIW. This water is formed by transforming the upwelling IPDW into lighter waters at the Southern Ocean surface, mostly through freshening by sea ice melt and precipitation (Abernathey et al., 2016; Haumann et al., 2016). The surface water experiences a substantial loss of biogenic CO_2_ to the atmosphere (Figure 2a) before being subducted as AAIW. Similarly to its anthropogenic component, these natural ventilation effects also reach into the IPDW layer (*γ*_N_ ∼ 27.8 kg m^−3^). The negative peak of $∆{PCO}_{2}^{res}$ just above the 27.8 kg m^−3^ isoneutral surface contributes to the sharp decline of ∆PCO_2_ just above the IPDW layer, but counteracts the decline below this layer (Figure 7, Table 2). Moreover, $∆{PCO}_{2}^{res}$ also includes air‐sea gas exchange effects related to DIC exchange at the surface that are the result of cooling or heating of a water mass at the surface before subduction. Such a signal of the so‐called solubility pump is visible in the positive values of the NADW core (Figure 6d). The arise from an uptake of carbon in the North Atlantic, driven by surface cooling, before the subduction of NADW to the deep ocean. This positive $∆{PCO}_{2}^{res}$ signature of NADW is lost southward within the Southern Ocean as these waters mix with waters ventilated in the Southern Ocean. Other residual effects included in $∆{PCO}_{2}^{res}$ arise from the choice of reference values, in particular ${sPO}_{4}^{ref}$, as discussed in Section 2.3. Overall, $∆{PCO}_{2}^{res}$ tends to increase ∆PCO_2_ below IPDW (Table 2) and therefore cannot explain the vertical ∆PCO_2_ maximum in IPDW.

#### 4.3.3 Vertical Difference Between DIC and ∆PCO_2_ Maxima Between 45° and 55°S

We now separately consider the effects of DIC and alkalinity on the vertical ∆PCO_2_ structure (Figures 7c and 7d) to better understand why ∆PCO_2_ decreases below the 27.8 kg m^−3^ isoneutral surface despite an equally high or higher carbon content (DIC) below this layer in the Southern Ocean (Figure 3). While the retained soft‐tissue and carbonate pumps both act to enhance the DIC storage in the interior ocean, the carbonate pump adds DIC to a greater depth than the retained soft‐tissue pump (Broecker & Peng, 1987; Kwon et al., 2011; Sarmiento et al., 1988). The difference between the vertical ∆PCO_2_ and DIC structure then largely results from the vertical alkalinity structure, since the retained soft‐tissue pump decreases the deep ocean alkalinity (increasing ∆PCO_2_) and the carbonate pump increases it (decreasing ∆PCO_2_). This impact of alkalinity on the zonal mean vertical ∆PCO_2_ structure between 45° and 55°S is shown in Figures 7c and 7d (see Section 2.4). Compared to the ∆PCO_2_ maximum of 175 ± 32 μatm in IPDW, ∆PCO_2_ in LCDW/NADW waters is 70 ± 46 μatm lower (Table 2). The largest contributor to this reduction, that is, 43%, is alkalinity, while the remainder is explained by a colder temperature (27%) and lower DIC (33%; Table 2; Figure 7d). The DIC contribution arises from low‐DIC NADW, which vanishes in depth coordinates, where alkalinity and temperature effects are the sole drivers of the vertical ∆PCO_2_ decline with depth (Figure 7c). These roles of alkalinity (61%) and temperature (49%) in driving a vertical ∆PCO_2_ decline become even larger when comparing the IPDW layer to AABW. Thus, the vertical DIC gradient cannot explain the lower ∆PCO_2_ in deeper and denser waters, except for the NADW core (*γ* ^N^ ∼ 28.05 kg m^−3^) that exhibits a DIC decrease with depth. In conclusion, the separation between the deeper DIC and shallower ∆PCO_2_ maxima is caused by the shallower depth of remineralization of organic carbon compared to the depth of dissolution of calcium carbonate that sets the relative contributions of DIC and alkalinity in driving ∆PCO_2_, reinforced by a decreasing temperature with depth.

## 5 Summary and Conclusions

We here show that the upwelling of high‐ΔPCO_2_ IPDW occurs along the 27.8 kg m^−3^ isoneutral surface in the Southern Ocean, which we identify as the major global pathway for old, pre‐industrial carbon to return to the surface and as the key source of Southern Ocean outgassing. The importance of this isopycnal and pathway for supply of old, nutrient‐rich IPDW to the surface of the Southern Ocean was previously identified through the location of the IPDW oxygen minimum (Talley, 2013). Here we highlight its relevance as the major return pathway of CO_2_ from the deep ocean to the atmosphere. This pathway draws from a high‐ΔPCO_2_ pool in the middepth northern Indo‐Pacific basins, which exceeds current atmospheric CO_2_ levels (400 μatm) by more than 800 μatm and is the result of a slow accumulation of carbon due to the remineralization of organic matter and its subsequent recirculation. The resulting high‐PCO_2_ IPDW propagates southward, where it subsequently upwells in the high‐latitude Southern Ocean, retaining a high‐ΔPCO_2_ signal that exceeds current atmospheric levels by 175 ± 32 μatm. Our findings thus provide observational evidence that there is a substantial transport of old, pre‐industrial CO_2_ from the deep ocean to the atmosphere through the Southern Ocean surface under present‐day conditions. Therefore, interior ocean measurements support the recent argument for a large natural CO_2_ release at the surface in the open waters around Antarctica (Bushinsky et al., 2019; Gray et al., 2018), sourced from relatively high PCO_2_ water that has upwelled to just beneath the mixed layer; this outgassing signal is best observed in winter when deep mixing entrains the high‐ΔPCO_2_ IPDW to the surface (Prend et al., 2022).

We find that the vertical subsurface ΔPCO_2_ gradient, and in particular the isopycnal that best characterizes its subsurface maximum, directly controls the meridional pattern of the Southern Ocean surface CO_2_ fluxes. The maximum outgassing between the Subantarctic Front and the winter‐time sea‐ice edge is caused by a circumpolar band of high‐ΔPCO_2_ IPDW just beneath the surface mixed layers. This characteristic ring pattern of Southern Ocean outgassing and high subsurface ΔPCO_2_ is due to the southward‐rising isopycnal surfaces that project the vertical ΔPCO_2_ maximum in IPDW onto the horizontal plane. The ring is circumpolar because the deep waters spiral southeastward with the ACC while they are rising, and are thus found all around Antarctica even though the original sources are localized boundary outflows from the Indian and Pacific basins (Tamsitt et al., 2017). Denser waters, such as NADW and the denser portion of IPDW (LCDW), reach the surface south of the maximum outgassing region in the ACC, and exhibit a much weaker outgassing, in part due to their lower ΔPCO_2_. Thus, the vertical ΔPCO_2_ distribution at mid‐latitudes directly explains the characteristic ring structure of Southern Ocean CO_2_ release (Figures 2a and 2e). Next to the source water properties investigated in this study, this pattern of CO_2_ release is further influenced by other surface processes, such as vertical mixing (Nicholson et al., 2022; Prend et al., 2022), solubility (Wu et al., 2019), biological production (Arteaga et al., 2019), and air‐sea gas exchange. In particular, storm driven mixing (Nicholson et al., 2022) and seasonal sea ice cover (Gupta et al., 2020; Loose & Schlosser, 2011; Rysgaard et al., 2011; Shadwick et al., 2021) might play an important role in modulating the seasonal rate of CO_2_ release.

Deep and surface ocean carbon content (DIC) are often used as a measure for potential Southern Ocean CO_2_ release. While the sources of elevated surface DIC in the Southern Ocean have been recently investigated (Wu et al., 2019), we here have shown that only considering DIC can lead to misinterpretations when it comes to surface CO_2_ fluxes, since the subsurface DIC structure critically differs from the subsurface ΔPCO_2_ structure that controls the surface CO_2_ fluxes. The DIC maximum occurs much deeper in the water column and remains high all the way to the bottom of the ocean, whereas ΔPCO_2_ peaks shallower and declines with depth. In our analysis, we show that the vertical distribution of ΔPCO_2_ and dissolved oxygen in the deep ocean are controlled primarily by the soft‐tissue pump, whereas the vertical distribution of DIC is influenced by both the retained soft‐tissue and the carbonate pumps, offsetting its maximum to deeper layers. This elevated carbon content (DIC) in the denser and deeper water masses, such as LCDW and AABW, is buffered by their carbon chemistry (high alkalinity) and colder temperatures, which limits CO_2_ outgassing from these waters. This reduced outgassing potential from higher alkalinity in the deeper waters is the result of a deeper depth of calcium carbonate dissolution compared to organic matter remineralization in the global ocean, which has been identified as a key mechanism for actively retaining carbon at depth over long time scales (Broecker & Peng, 1987; Hain et al., 2010; Krumhardt et al., 2020; Kwon et al., 2011; Toggweiler, 1999). Consequently, next to adequate ocean circulation and gas‐exchange kinetics, a realistic representation of the vertical remineralization and dissolution profiles in the global ocean is an important pre‐requisite for Earth System Models to accurately simulate Southern Ocean CO_2_ fluxes and the global ocean carbon cycle, as well as their response to climatic changes (Kwon et al., 2009).

Model simulations project an enhanced future upwelling of deep waters in the Southern Ocean (Downes & Hogg, 2013) due to a poleward intensification of westerly winds (Bracegirdle et al., 2020) and changes in the surface buoyancy forcing (Bishop et al., 2016; Downes et al., 2018). Such increased upwelling could substantially amplify the leakage of pre‐industrial CO_2_ from the deep ocean (Lovenduski et al., 2007; Toggweiler & Russell, 2008) and lead to a “saturation” of the anthropogenic CO_2_ uptake by the ocean (Le Quéré et al., 2007; Lovenduski et al., 2007). However, there is still limited confidence in future projections of upwelling in the Southern Ocean (Meredith et al., 2019) since these models do not resolve mesoscale eddies (Bishop et al., 2016; Meredith et al., 2012; Morrison & Hogg, 2013) that are important for the ocean's carbon transport (Abernathey & Ferreira, 2015; Dufour et al., 2015) and suffer from large biases in their water mass structure (Beadling et al., 2020; Downes et al., 2018). In addition, global climate models struggle to produce the correct patterns, magnitudes (Lenton et al., 2013; Mongwe et al., 2018), and temporal variability (Gruber et al., 2019) of surface CO_2_ fluxes in the Southern Ocean. Observational evidence derived from chlorofluorocarbon measurements points toward an increased upwelling since the early 1990s (Ting & Holzer, 2017; Waugh et al., 2013). However, recent findings also show that the relation between upwelling deep waters and changes in the surface CO_2_ fluxes is much more complex and exhibits strong fluctuations on decadal time scales (DeVries et al., 2019; Landschützer et al., 2015). Our results imply that not only the strength of circulation and ventilation changes in the Southern Ocean play an important role in altering the release of CO_2_ from the deep ocean to the atmosphere, but also the depth level from which waters are upwelled. Moreover, they also imply that changes in subsurface carbon chemistry could impact the CO_2_ release from the deep ocean. These implications of our results highlight the importance of improving the subsurface carbon chemistry, water‐mass structure, and circulation in global climate models in order to better assess future changes in atmospheric CO_2_ in response to ocean circulation changes.

## Supporting information

Supporting Information S1

## Data Availability

All data and tools underlying this analysis are openly available: Mapped Global Ocean Data Analysis Project version 2 (https://www.glodap.info/index.php/mapped-data-product; https://doi.org/10.5194/essd-8-325-2016); Southern Ocean Carbon and Climate Observations and Modeling float data (Snapshot 2021‐05‐05; https://doi.org/10.6075/J0T43SZG); globally mapped CO_2_ flux estimate based on the Surface Ocean CO_2_ Atlas Database and Southern Ocean Carbon and Climate Observations and Modeling biogeochemistry floats (1982–2017, NCEI Accession 0191304, Version 2.2; https://doi.org/10.25921/9hsn-xq82); Global monthly gridded sea surface pCO_2_ product from 1982 onward and its monthly climatology (NCEI Accession 0160558, Version 5.5; https://doi.org/10.7289/V5Z899N6); CDIAC CO_2_ flux estimated from air‐sea difference in CO_2_ partial pressure (revised October 2009; https://www.ldeo.columbia.edu/res/pi/CO2/carbondioxide/pages/air_sea_flux_2010.html); MATLAB Program Developed for CO_2_ System Calculations (https://doi.org/10.3334/CDIAC/otg.CO2SYS_MATLAB_v1.1); and NOAA/NSIDC Climate Data Record of Passive Microwave Sea Ice Concentration (Version 3, 1979–2018; https://doi.org/10.7265/N59P2ZTG).
