# Supplementary material for: The Deep Ocean's Carbon Exhaust
Source: Global Biogeochem Cycles. 2022 Jul 21;36(7):e2021GB007156. doi: 10.1029/2021GB007156 (PMC9540790; doi:10.1029/2021GB007156)
Supplement: Supplementary file 1 — Supporting Information S1 [file GBC-36-e2021GB007156-s001.docx]

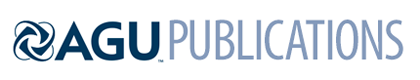


*Global Biogeochemical Cycles*

Supporting Information for

**The Deep Ocean’s Carbon Exhaust**

Haidi Chen^1^, F. Alexander Haumann^1*^, Lynne D. Talley^2^, Kenneth S. Johnson^3^, and Jorge L. Sarmiento^1^

^1^Atmospheric and Oceanic Sciences Program, Princeton University, Princeton, NJ, USA.

^2^Scripps Institution of Oceanography, University of California, San Diego, La Jolla, California, USA.

^3^Monterey Bay Aquarium Research Institute, Moss Landing, California, USA

*Corresponding author: F. Alexander Haumann (alexander.haumann@gmail.com)

**Contents of this file**

Figures S1

**Introduction**

This Supporting Information includes Figure S1. It shows a sensitivity test of the estimated soft-tissue pump potential partial pressure of carbon-dioxide (∆PCO_2_^soft^) and residual ∆PCO_2_ (∆PCO_2_^res^) in the Pacific basin to different phosphate (PO_4_) reference values used in the calculation.


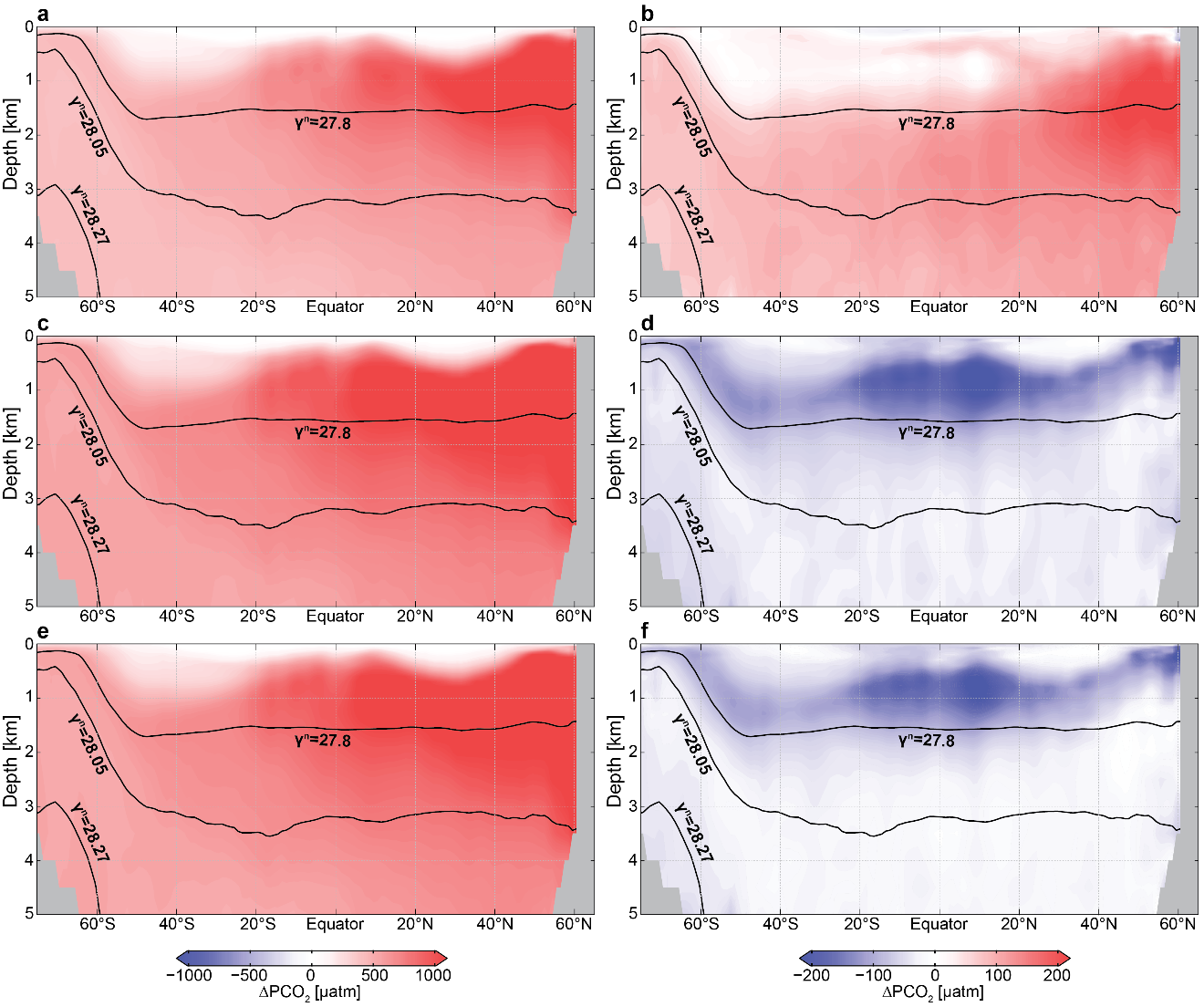


Figure S1. Sensitivity of the calculated zonal mean ∆PCO_2_^soft^ (a, c, e) and ∆PCO_2_^res^ (b,d,f) in the Pacific basin to different PO_4_ reference values. a,b, ∆PCO_2_ using the mean present day surface PO_4_ reference value of 0.47 µmol kg^−1^. c,d, ∆PCO_2_ using the PO_4_ reference value of 0.07 µmol kg^−1^ used by Gruber & Sarmiento (2002). e,f, ∆PCO_2_ using the PO_4_ reference value of 0.1 µmol kg^−1^ used in this study.

References

Gruber, N., & Sarmiento, J. L. (2002). Large-scale biogeochemical–physical interactions in elemental cycles. *Biological–Physical Interactions in the Sea,* A. R. Robinson, J. J. McCarthy, and B. J. Rothschild, Eds., *The Sea: Ideas and Observations on Progress in the Study of the Seas,* Vol. 12, John Wiley & Sons, 337–399.
